# Applicability and added value of novel methods to improve drug development in rare diseases

**DOI:** 10.1186/s13023-018-0925-0

**Published:** 2018-11-12

**Authors:** Marian Mitroiu, Katrien Oude Rengerink, Caridad Pontes, Aranzazu Sancho, Roser Vives, Stella Pesiou, Juan Manuel Fontanet, Ferran Torres, Stavros Nikolakopoulos, Konstantinos Pateras, Gerd Rosenkranz, Martin Posch, Susanne Urach, Robin Ristl, Armin Koch, Spineli Loukia, Johanna H. van der Lee, Kit C. B. Roes

**Affiliations:** 1 Clinical Trial Methodology, Julius Center for Health Sciences and Primary Care, Biostatistics and Research Support, University Medical Center Utrecht, University of Utrecht, Heidelberglaan 100, 3584 CX Utrecht, The Netherlands; 2 Departament de Farmacologia, de Terapèutica i de Toxicologia, Universitat Autònoma de Barcelona, Unitat Docent Parc Taulí, c/ Parc Taulí 1, 08208 Sabadell, Spain; 3 Clinical Pharmacology Department, Research Institute Puerta de Hierro, C/Manuel de Falla, 1, 28222 Majadahonda, Madrid, Spain; 4 Unitat de Farmacologia Clínica, Hospital de Sabadell, Institut d’Investigació i Innovació Parc Taulí I3PT - Universitat Autònoma de Barcelona, c/ Parc Taulí 1, 08208 Sabadell, Spain; 5 Departament de Farmacologia, de Terapèutica i de Toxicologia, Universitat Autònoma de Barcelona, Hospital de Sant Pau, C/St Antoni Maria Claret 167, 08025 Barcelona, Spain; 6 Biostatistics Unit, Faculty of Medicine, Universitat Autònoma de Barcelona, 08193 Bellaterra, Barcelona, Spain; 7 Medical Statistics Core Facility, IDIBAPS - Hospital Clinic Barcelona, C/Mallorca 183, Floor -1, 08036 Barcelona, Spain; 8 Section for Medical Statistics, Center for Medical Statistics, Informatics, and Intelligent Systems, Medical University of Vienna, Spitalgasse 23, 1090 Vienna, Austria; 9 Hannover Medical School, Carl-Neuberg-Str. 1, 30625 Hannover, Germany; 10 Paediatric Clinical Research Office, Woman-Child Center, Academic Medical Center, University of Amsterdam, Amsterdam, the Netherlands

**Keywords:** Orphan, Rare condition, Clinical trials, Small population, Statistical methods

## Abstract

**Background:**

The ASTERIX project developed a number of novel methods suited to study small populations. The objective of this exercise was to evaluate the applicability and added value of novel methods to improve drug development in small populations, using real world drug development programmes as reported in European Public Assessment Reports.

**Methods:**

The applicability and added value of thirteen novel methods developed within ASTERIX were evaluated using data from 26 European Public Assessment Reports (EPARs) for orphan medicinal products, representative of rare medical conditions as predefined through six clusters. The novel methods included were ‘innovative trial designs’ (six methods), ‘level of evidence’ (one method), ‘study endpoints and statistical analysis’ (four methods), and ‘meta-analysis’ (two methods) and they were selected from the methods developed within ASTERIX based on their novelty; methods that discussed already available and applied strategies were not included for the purpose of this validation exercise. Pre-requisites for application in a study were systematized for each method, and for each main study in the selected EPARs it was assessed if all pre-requisites were met. This direct applicability using the actual study design was firstly assessed. Secondary, applicability and added value were explored allowing changes to study objectives and design, but without deviating from the context of the drug development plan. We evaluated whether differences in applicability and added value could be observed between the six predefined condition clusters.

**Results and discussion:**

Direct applicability of novel methods appeared to be limited to specific selected cases. The applicability and added value of novel methods increased substantially when changes to the study setting within the context of drug development were allowed. In this setting, novel methods for extrapolation, sample size re-assessment, multi-armed trials, optimal sequential design for small sample sizes, Bayesian sample size re-estimation, dynamic borrowing through power priors and fall-back tests for co-primary endpoints showed most promise - applicable in more than 40% of evaluated EPARs in all clusters. Most of the novel methods were applicable to conditions in the cluster of chronic and progressive conditions, involving multiple systems/organs. Relatively fewer methods were applicable to acute conditions with single episodes. For the chronic clusters, Goal Attainment Scaling was found to be particularly applicable as opposed to other (non-chronic) clusters.

**Conclusion:**

Novel methods as developed in ASTERIX can improve drug development programs. Achieving optimal added value of these novel methods often requires consideration of the entire drug development program, rather than reconsideration of methods for a specific trial. The novel methods tested were mostly applicable in chronic conditions, and acute conditions with recurrent episodes.

**Electronic supplementary material:**

The online version of this article (10.1186/s13023-018-0925-0) contains supplementary material, which is available to authorized users.

## Background

### Background on ASTERIX project

ASTERIX was a novel EU-funded research project focusing on the development of more efficient and effective research designs to study new drugs and treatments for rare diseases. The overall aim was to achieve more reliable and cost-efficient clinical development of treatments for rare diseases and to stimulate the search for treatments for these devastating and largely ignored diseases.

The main objectives were to:

- Develop design and analysis methods for single trials and series of trials in small populations.
- Include patient-level information and perspectives in design and decision making throughout the clinical trial process.
- Validate new methods and propose improvements for regulatory purposes.

ASTERIX worked through six highly interactive and interdependent Work Packages ranging from development of methodology, stakeholder participation to the dissemination of the results. Unique in this project was that patients were directly involved in the research process and their input is taken into account in design and analysis of studies.

### Context

Six percent of the global population is affected by one of the estimated 5000–8000 rare diseases at some stage in their life [1]. In Europe a disease is classified as ‘rare’ if it affects less than 5 in 10,000 people [2]. Evaluating interventions aimed at preventing, diagnosing or treating a rare disease is a challenge, and can lead to slow evaluation and approval of Orphan Medicinal Products (OMPs) for marketing, and thereby delay access by patients [3]. To stimulate the development of medicines for rare diseases the EU Orphan Regulation came into effect in 2000. This regulation provides an incentive for research, development and marketing of OMPs to target rare diseases [4]. Although more than 1800 orphan drug designations have been granted since 2000, by 2017 only 129 OMPs were granted market authorisation [5]. Hence, although drugs do become available, a treatment still needs to be found for the vast majority of rare diseases. The main issue that distinguishes medicines development for rare diseases from more common diseases is the challenge of generating robust clinical evidence. The limited recruitment potential calls for an efficient study design, able to estimate the treatment effect in a valid and reliable way with a small number of patients [6].

There is an abundance of methodology to improve the design and analysis of individual trials, often essentially aimed at increasing efficiency: extract more information from the same trial, increase the probability of success of an individual clinical trial and enable the conduct of smaller trials. Yet, progress for clinical trials in truly small populations has proven difficult to achieve. Some frameworks have been proposed to guide the choice of the best suited methodology and study designs in drug development for such rare diseases. At the regulatory level, the European Medicines Agency (EMA) released the ‘Guideline on Clinical Trials in Small Populations’, which summarises a range of possible approaches in the context of small populations in drug development, acknowledging that any efficiency improvements for small population clinical trials would also be relevant to larger trials and vice-versa [7]. Other available frameworks typically aimed to propose algorithms or decision processes to arrive at the most suited design for a given clinical trial. These focus either on a specific condition ([8, 9]), a specific method or group of methods ([10, 11]), or provide general recommendations [12–15].

However, most of these algorithms or frameworks are guided by items related to only a few characteristics of the condition such as clinical course, timing and reversibility of the outcome, or trial feasibility, and they are not always exhaustive to fit all possible situations.

ASTERIX was a novel EU-funded research project (7th Framework Program (FP7) Call – Health.2013.4.2–3) focusing on the development of more efficient and effective research designs to study new drugs and treatments for rare diseases. The overall aim was to achieve more reliable and cost-efficient clinical development of treatments for rare diseases and to stimulate the search for treatments for these devastating and largely ignored diseases. ASTERIX decided to focus on progress in clinical research for new treatments for rare diseases. The vision of ASTERIX was that such progress can be best made, by advancing in coherence: (1) statistical methodology for design and analysis, (2) incorporation of the patient perspective in design and outcomes and (3) uptake in practice and regulatory guidance [16].

Within the ASTERIX project 13 novel methods have been developed proposing innovative approaches to adapt and analyse clinical trials on small populations and rare diseases (Table 1). We aimed to evaluate these methods for added value against an appropriate framework to guide application, preferably tailored to characteristics of the medical condition. The limitations of the existing frameworks to provide guidance that directly incorporates characteristics of the medical condition treated are obvious. Apart from their low prevalence, orphan diseases are a highly heterogeneous group of diseases. Such heterogeneity makes it very difficult to issue useful regulatory recommendations relevant to all (or at least most) possible clinical situations in the course of uncommon diseases. Nevertheless, some groups of conditions share similar clinical characteristics linked to the applicability of certain trial designs and general approaches.

**Table 1:** Overview of the methods that were evaluated

Description of the method	Requirements for use of the method	Potential advantages	Potential disadvantages
		compared to developmental plans that supported approval	
LEVEL OF EVIDENCE			
Extrapolation [50]			
In small populations, a full independent drug development program to demonstrate efficacy may not be ethical, feasible or necessary. Extrapolations of evidence from a larger population to the smaller target population is widely used to support decisions in this situation.For the justification of requirements specified in EMA Paediatric Investigation Plans, this paper discusses how to specify the clinical trial design in the target population, when the data from the source population at the time of planning is not available but development in the target population will only start, after a treatment effect in the source population has been demonstrated. A framework based on prior beliefs is formulated to investigate whether the significance level for the test of the primary endpoint in confirmatory trials can be relaxed, and the sample size reduced, while controlling a certain level of certainty about the effects. The procedure is based on a so called skepticism factor, that quantifies the belief that a treatment effect observed in the larger population can be extrapolated to the target population.	Factors that influence the possibility for extrapolation: ▪ Same underlying mechanism of action, similarity of response to treatment, similar dose-response relationship so the mechanism is translatable to the target population ▪ Same disease symptoms in adults and children, regarding similarity of disease progression ▪ Timing of the paediatric trial compared to the adult trial should allow extrapolation ▪ Repurposed drug or not or extension of indication	▪ Optimised use of available evidence for the entire development programme ▪ Reduction of sample size	▪ Difficulty lies in its novelty and application ▪ Parameters for prior need to be set realistically
META-ANALYSIS			
Prior distributions for variance parameters in sparse-event meta-analysis (Pateras K, personnal communication)			
The small sample sizes in rare diseases make it particularly valuable to pool the data of small studies in a meta-analysis. When the primary outcome is binary, small sample sizes increase the chance of observing zero events. The frequentist random-effects model is known to induce bias and to result in improper interval estimation of the overall treatment effect in a meta-analysis with zero events. Bayesian hierarchical modelling could be a promising alternative. Bayesian models are known for being sensitive to the choice of between-study variance (heterogeneity) prior distributions in sparse settings. In a rare disease setting, only limited data will be available to base our prior on, therefore, the need to identify priors with robust properties is crucial. This paper shows that the Uniform (− 10; 10) heterogeneity prior on the log (T2) scale shows appropriate 95% coverage and induces relatively acceptable under/over estimation of both the overall treatment effect and heterogeneity, across a wide range of heterogeneity levels. We illustrate the results with two examples of a meta-analyses with a few small trials.	▪ > = 2 RCTs ▪ Same endpoint in at least two trials, from which one primary endpoint ▪ Binary endpoint(s) ▪ Sparse events ▪ Not prerequisite but patients allocation ratio ideally 1:1 ▪ Treatment effect size estimates reported in harmonized (or harmonisable) manner ▪ Not prerequisite but ideally equally allocated number of patients per study	▪ Optimised use and variance estimation in a sparse-event meta-analysis ▪ Quicker, optimal selection and use of appropriate heterogeneity priors distributions	▪ Use of informative priors (even for heterogeneity) may be controversial. ▪ Optimal choice is simulation based, and unknown if it is best in a specific situation
Heterogeneity estimators in zero cells meta-analysis [51]			
When a meta-analysis consists of a few small trials that report zero events, accounting for heterogeneity in the estimation of the overall effect is challenging. In practice, the data poses restrictions on the meta-analysis method employed that lead to deviations from the pre-planned analysis, such as the presence of zero events in at least one study arm. Estimators that performed modestly robust when estimating the overall treatment effect across a range of heterogeneity assumptions were the Sidik-Jonkman, Hartung-Makambi and improved Paul-Mandel. The relative performance of estimators did not materially differ between making a predefined or data-driven choice. The simulations confirmed that heterogeneity cannot be estimated reliably in a few small trials that report zero events. Estimators whose performance depends strongly on the presence of heterogeneity should be avoided. The choice of estimator does not need to depend on whether or not zero cells are observed.		▪ Quicker and optimal selection of heterogeneity estimator in a sparse-event meta-analysis	▪ Niche method and does not cover all heterogeneity estimators
INNOVATIVE TRIAL DESIGNS			
Critical appraisal of delayed-start design proposed as alternative to randomized controlled design in the field of rare diseases [52]			
In a delayed start randomization design, patients are randomised at baseline to receive either the intervention (early-start group) or placebo (delayed-start group) and after a certain period of time, the latter switch to the intervention until trial completion, therefore, reducing the time in placebo. Data collected at the end of placebo-phase allow for causal inferences, whereas the data collected at trial completion allow for investigation of disease-modifying effects.	▪ The comparator needs to be placebo (severity and predictability of condition to allow for placebo arm use) ▪ Intervention needs to have lasting response/remission ▪ Ideal for slowly and constant progressive diseases	▪ All patients eventually receive treatment ▪ Robust evidence from the randomised and controlled first phase of the design ▪ The switch time-point can provide extra information	▪ Delay in some patients receiving treatment, compared to a single arm trial (but not different from parallel control arm) ▪ Not (always) more efficient than parallel arm trial
Sample size reassessment and hypothesis testing in adaptive survival trials [53]			
This design allows a sample size reassessment during a trial where the primary outcome is the time to the occurrence of an event. The sample size reassessment is performed in an interim analysis and may be based on unblinded interim data, including secondary endpoints. This paper discusses major drawbacks of a fully unmasked sample-size recalculation, i.e. a decision based on all available efficacy and safety data, are potential intentional changes in the behaviour of the investigators, and the potential impossibility to include all patients in the final analysis and propose a test statistic for inclusion of all patients.	▪ In case the sample size re-assessment is unmasked ▪ Time to outcome faster than accrual rate ▪ At least one interim analysis	▪ Increased precision for sample size reassessment ▪ Preservation of type I error ▪ Inclusion of all (more patients) in the final test statistic (increases regulatory acceptability)	▪ Logistically resource-wise more demanding ▪ Risks of bias associated with unblinded re-assessment
Multi-arm group sequential designs with a simultaneous stopping rule [54]			
A design with 3 arms or more, with planned interim analyses with a simultaneous stopping rule using predefined boundaries. This rule aims to detect at least one efficacious treatment out of all tested arms. The trial may stop for one or more arms because of futility, or for all arms when efficacy is proven for at least one of them.	▪ At least 3 arms including control (placebo) ▪ At least 1 interim analysis ▪ Time to outcome faster than accrual/enrolment rate Developed for normally distributed endpoints, conferrable to other types (i.e. binary), relying on asymptotic normality of the corresponding test statistics.	▪ More patients are randomized to a treatment arm due to the common control arm ▪ More efficient use of available patients (i.e. lower average sample number than for the separate stopping rule, up to 21% depending on design): ▪ Possibility of head to head comparisons between different treatments	▪ Not applicable to historically/externally controlled studies ▪ More complex trial conduct ▪ In case of no early estopping, interim analyses could result in an overall longer trial compared to single stage designs ▪ The potential to marginally miss a second efficacious intervention
Sequential design for small samples starting from a maximum sample size [55]			
Using a group sequential design, an analysis will be performed before the trial is finished, based on the available data collected at that (pre-defined) moment. The aim of this design is to pick up large benefits or lack of benefit signals earlier. The proposed method uses the maximum available sample size as a starting point for planning the study, taking into account the desired chance to pick up a therapeutic effect if it really exists, and then continues with the refined calculations of the limit boundaries. This method determines the optimal number of interim analyses to be performed, while keeping the chance low of concluding that a treatment works - while in real life it does not work.	▪ Needs to start from maximum sample size that can be recruited ▪ At least 1 interim analysis ▪ Time to outcome faster than accrual rate	▪ Increased precision when using prior knowledge (from historical data or previous trials) to estimate treatment effect size, and thereby increased precision for the adjustment of boundaries ▪ Optimised use of maximum available patient pool in the development programme (especially for ultra-rare settings)	▪ More interim analyses will provide extra work ▪ Sufficient level of evidence, but not overwhelming
Bayesian sample size re-estimation using power priors [56]			
Bayesian statistics, use probability distributions, often including a probability of the belief in the intervention before the start of the trial (the prior). For normally distributed outcomes, an assumption for the variance needs to be made to inform the sample size needed, which is usually based on limited prior information, especially in small populations. When using a Bayesian approach, the aggregation of prior information on the variance with newly collected data is more formalized. The uncertainty surrounding prior estimates can be modelled with prior distributions. The authors adapt the previously suggested methodology to facilitate sample size re-estimation. In, addition, they suggest the employment of power priors in order for operational characteristics to be controlled.	▪ At least 1 interim analysis ▪ Randomisation ▪ 1 control and 1 experimental arm ▪ Developed for continuous endpoints, transportable to other types of outcomes	▪ More efficient use of available patients for the development programme (i.e. smaller sample size) ▪ Increased precision when optimally using prior knowledge (from historical data or previous trials) to estimate treatment effect size ▪ Control of type I error	▪ Extra patients needed in case of effect size overestimation
Dynamic borrowing using power priors that control type I error [57]			
In rare diseases, where available data is scarce and heterogeneity between trials is less well understood, the current methods of meta-analysis fall short. The concept of power priors can be useful, particularly for borrowing evidence from a single historical study. Such power priors are expressed as a parameter, which in most situations has a direct translation as a fraction of the sample size of the historical study that is included in the analysis of the new study. However, the possibility of borrowing data from a historical trial will usually be associated with an inflation of the type I error. Therefore in this paper a new, simple method of estimating the power parameter in the power prior formulation is suggested, suitable when only one historical dataset is available. This method is based on predictive distributions and parameterized in such a way that the type I error can be controlled, by calibrating the degree of similarity between the new and historical data.	▪ Essential to have robust data from ideally previous similar studies ▪ Developed for normal responses in a one or two group setting, but the generalization to other models is straightforward	▪ More efficient use of available patients for the development programme (i.e. smaller sample size) ▪ Increased precision when optimally using prior knowledge (from historical data or previous trials) to estimate treatment effect size ▪ Control of operational characteristics while modelling the heterogeneity between the historical and emerging data	▪ Extra patients needed in case of effect size overestimation
STUDY ENDPOINTS AND STATISTICAL ANALYSIS			
Fallback tests for co-primary endpoints [58]			
Usually, when the efficacy of an intervention is measured by co-primary endpoints, efficacy may be claimed only if for each endpoint an individual statistical test is significant. While this strategy controls the type I error, it is often very conservative, and does not allow for inference if only one of the co-primary endpoints shows significance.This paper describes the use of fall-back tests. They reject the null hypothesis in exactly the same way as the classical tests, with the advantage that they allow for inference in settings where only some of the co-primary endpoints show a significant effect. Similarly to the fall-back tests defined for hierarchical testing procedures, these fall-back tests for co-primary endpoints allow to continue testing, even the primary objective of the trial was not met.	▪ At least 2 co-primary endpoints ▪ One test per endpoint	▪ No need for hierarchical pre-specification and testing of multiple co-primary endpoints ▪ Improved statistical testing (more chances to detect one dimension of treatment effect and benefit even if the primary objective has not been met) ▪ Control of family-wise error rate (FWER)	▪ Potentially more patients needed
Optimal exact tests for multiple binary endpoints [59]			
In confirmatory trials with small sample sizes, hypothesis tests developed for large samples - based on asymptotic distributions - are often not valid. Exact non-parametric procedures are applied instead. However, exact non-parametric procedures are based on discrete test statistics and can become very conservative. With standard adjustments for multiple testing, they become even more conservative. Exact multiple testing procedures are proposed, for the setting where multiple binary endpoints are compared in two parallel groups. Based on the joint conditional distribution of the test statistics of Fisher’s exact test, the optimal rejection regions for intersection hypothesis tests are constructed. To efficiently search the large space of possible rejection regions, the an optimization algorithm is proposed based on constrained optimization and integer linear programming. Depending on the objective of the optimization, the optimal test yields maximal power under a specific alternative, maximal exhaustion of the nominal type I error rate, or the largest possible rejection region controlling the type I error rate. Applying the closed testing principle, the authors construct optimized multiple testing procedures with strong familywise error rate control. In addition, they propose a greedy algorithm for nearly optimal tests, which is computationally more efficient.	▪ Multiple dichotomous/binary outcomes ▪ Two or more endpoints ▪ Gain is strongest in very small sample sizes (1 to 50 per group) ▪ A priori definition of the optimization criterion. ▪ Prior assumption on effect sizes when optimizing power	▪ Optimised multiple testing procedure for dichotomous endpoints ▪ Maximal power use of the statistical test ▪ Control of family-wise error rate (FWER) ▪ Robust evidence ▪ Useful for (very) small sample sizes	▪ Potentially more patients needed
Simultaneous inference for multiple marginal GEE models [60]			
A framework is proposed for using generalized estimating equation models for each endpoint marginally considering dependencies within the same subject. The asymptotic joint normality of the stacked vector of marginal estimating equations is used to derive Wald-type simultaneous confidence intervals and hypothesis tests for linear contrasts of regression coefficients of the multiple marginal models. The small sample performance of this approach is improved by adapting the bias correction proposed by Mancl and DeRouen to the estimate of the joint covariance matrix of the regression coefficients from multiple models. As a further improvement a multivariate t-distribution with appropriate degrees of freedom is specified as reference distribution. Alternatively, a generalized score test based on the stacked whom correspondence should be addressed estimating equations is derived. By means of simulation studies, control of type I error rate for these methods is shown even with small sample sizes and also increased power compared to a Bonferroni multiplicity adjustment. The proposed methods are suitable to efficiently use the information from dependent observations of multiple endpoints in small-sample studies. If simultaneous confidence intervals for two or more endpoints are of interest, this approach can be used. Additionally, an R software package has been developed (`mmmgee’) for computational implementation of this framework.	▪ Repeated measurements	▪ Robust evidence from longitudinal data ▪ Estimation of endpoints separately while taking into account dependencies within the same patient	▪ Technically more complex ▪ Not all trials make use of repeated measurements
Goal Attainment Scaling [61]			
Goal Attainment Scaling is a measurement instrument that measures the attainment of different goals of patients in a standardized way. The goals are measured in the same way for every patient, but the content of the goals can be different between patients. To apply goal attainment scaling, the caregiver and the patient sit together to decide what the goals of the patient are, and how they can be defined in five levels. Next, the patient receives the intervention (preferably blinded). Then after the intervention the patient and doctor assess how well the goals have been attained. Due to the different content of the goals for different patients goal attainment scaling can be used in groups of patients who all have different complaints, which is often the case in rare diseases. Another advantage is that it is very sensitive to change.	▪ Essential that there is no primary endpoint that is relevant for all patients ▪ Heterogeneous disease course with stable baseline values for goal(s) setting ▪ It has to be actual treatment (not prevention) ▪ (Can only be interpreted in a) randomised controlled trial ▪ Measurement relevant at functional level	▪ The goals are individually defined in consultation with patients and chosen per patient, hence customised measurement of therapeutic effect ▪ Time-demanding aspect, needed for detailed construction and definition of goals, may be less of a concern when there is a (very) limited number of available patients ▪ Direct patient involvement in efficacy assessment	▪ Time-consuming to set (multiple) goals individually per patient ▪ Choice of goals must be realistic and associated with potential treatment effect ▪ Translation of effect size at group level to clinical benefit difficult

Thus, within the ASTERIX project we used a heuristic framework that could help identify groups of medical conditions – defined as the combination of clinical situation and a given therapeutic approach to be tested - for which similar methods could be useful for drug development. The six condition clusters were used to check for patterns within clusters of conditions that share in common similar features. These are reflected also in methodological and trial design challenges. The clusters were used as a strategy to try and provide methodological insights by cluster of conditions and overcome the challenges due to the large number and high variation of rare conditions. The methodology, reasoning and derivation behind the conditions clustering is detailed in ([17], to appear).

In this study, we aimed to evaluate the applicability and added value (via the potential advantages) of the 13 novel methods developed within the ASTERIX project against a comprehensive set of real life examples of drug development programs for OMPs, as identified in European Public Assessment Reports (EPARs). The framework of medical condition clusters was applied as a way to structure our evaluation, so that guidance on the (novel) methods could be given more specifically at the condition cluster level. In addition, we described advantages and disadvantages of using the newly developed methodology. Based on the applicability and potential advantages and disadvantages, we aimed to tailor guidance on the use of this new methodology to specific medical condition clusters.

## Methods

We included all novel methods that were developed within the ASTERIX project and that had been reported in a published or (nearly) submitted manuscript by 1 September 2017.

There are numerous other methods and tools available that address challenges encountered in conducting research for rare diseases and small populations (i.e. n-of-1 trials, patient registries), and some were investigated in ASTERIX [18]. However, for the purpose of this research we focused only on the novel methods developed within ASTERIX. We excluded manuscripts that discussed already existing methods, or described a new perspective on an already existing method. We categorised the methods into four main groups:

- Six ‘innovative trial designs’, including: delayed-start design, a method for interim analysis and stopping rules in multi-arm parallel trials, two methods for sample-size reassessment (one for adaptive survival trials, and a second one with a Bayesian approach for continuous end-points), a method to optimize boundaries in group-sequential designs, and a method to weight prior information in Bayesian trials based on similarity of previous data.
- One ‘level of evidence’ method, consisting of a set of recommendations to check if prior information can be used for inference allowing to relax the significance level in confirmatory trials, reducing sample size while controlling for certainty.
- Four ‘study endpoints and statistical analysis’ methods, including: three methods to analyze multiple end-points (one for analysis of repeated measurements of multiple end-points, one allowing conclusions for multiple co-primary endpoints even when not all meet statistical significance, and an exact non-parametric method for multiple binary end-points), and a patient-centered measurement instrument (Goal Attainment Scale or GAS) aimed to standardise individual patients’ functional outcomes in conditions with heterogeneous clinical expression.
- Two ‘meta-analysis’ methods, both aimed at improving the management of heterogeneity estimators in meta-analysis of sparse-event studies.

### Heuristic framework used

Six clusters of medical conditions were defined: ‘acute single episodes’, ‘conditions with acute recurrent episodes’, ‘chronic condition with stable or slow progression’, ‘chronic progressive condition, led by one system/organ’, ‘chronic progressive condition led by multiple systems/organs’ and ‘chronic staged conditions’ (Additional file 1: Appendix 1). In addition to this classification, a consideration of extreme rarity was also taken into account, since ultra-rare conditions (< 1/100,000) have additional limitations to the recruitment potential.

### Selection of EPARs for validation

We selected 26 EPARs out of the available 125 OMPs approved by EMA between 2001 (start of the Orphan Regulation application) and 2014 (time cut-off when this research was initiated). We aimed to select EPARs that represent the conditions within each of the six medical condition clusters, without prior information about the potential applicability of the novel developed methods. We used the following criteria for selection:

- In principle four EPARs for each condition cluster. We considered this number sufficient to capture the diversity within the cluster, but acknowledged that exceptions are still possible;
- Since extreme rarity of a given medical condition raises additional limitations to the recruitment potential, for each condition cluster at least one EPAR describing an ultra-rare condition (affecting < 1/100,000 persons in the EU) was selected;
- Per medical condition we included only one EPAR. The same drug could have been included more than once if developed for more than one indication, although none actually was;
  At least one repurposed drug per cluster, defined as a drug that was already in use for a different medical condition and for which a new authorization was applied and granted for an orphan indication. Repurposed drugs may have different development approaches because part of the already available information may be extrapolated from former use to the new application.

If information in EPARs was insufficiently detailed, FDA summary basis of approval [19], published original articles and public clinical trial registries were consulted in order to obtain the necessary information for assessment [20–22].

### Method of evaluation of applicability and added value of novel methodology

Key characteristics of the studies that were used as pivotal evidence to support approval of orphan products were extracted from the EPARs and systematised (Additional file 1: Appendix 3). EPARs were used as the basic source for the data extraction since they contained the key information for the regulatory assessment in the EU. However, when EPARs were insufficiently detailed (i.e. recruitment pattern or recruitment timing was missing, etc.), we investigated other publicly available sources, such as the reports from FDA. A data extraction form was created, pilot tested and in multiple iterations refined by discussion amongst nine reviewers (Additional file  1: Appendix 1). One researcher (MM) extracted the key characteristics from the studies reported in the EPARs. One researcher extracted the pre-requisites of the methods, checked by a second researcher (KOR) and the (co)developer of the method. Five researchers summarized the OMPs and orphan conditions. The summaries and extracted data were also checked by at least one researcher independent of the previous tasks during the evaluation of the applicability of the methods.

To judge on applicability, at least two researchers were involved: 1) MM or KOR or both, and 2) the (co)developer of the method evaluated. In approximately a quarter of the cases - and in all cases with any uncertainties or unclarities - the applicability evaluation was discussed with the ASTERIX project lead. If opinions did not concur, agreement was reached in a discussion between the researchers who judged on applicability, (co)developers of the methods and the ASTERIX project lead. We summarised the key features of the 13 novel ASTERIX methods, their prerequisites and potential advantages and disadvantages*.* Advantages and disadvantages of the methods were extracted from the papers and manuscripts, if reported. When not reported, advantages and disadvantages were added by the reviewers (MM and KOR), based on logical reasoning. These were reviewed by the (co-)developers of the methods, and refined where necessary to reflect the advantages/disadvantages of applying the particular method. We evaluated these prerequisites against the design (characteristics) of the pivotal studies (main efficacy studies that supported the regulatory evaluation and approval) and characteristics of the orphan conditions mirroring the methods’ prerequisites and design elements in a pilot including four studies reported in two EPARs (for OMPs Savene and Cayston [23]), and then we refined the list of characteristics with study and applicability details (Additional file 1: Appendix 1). In addition, we evaluated whether the method (if applicable) would have added value compared to the currently used method. We used the currently used method – rather than using one common standard as comparator as it would be difficult to have a standard given the plethora of challenges associated with each condition and patient population. To our opinion, this comparison best reflects the improvements that can be achieved for each scenario. The extracts, interpretation and conclusions were sent for validation to the lead authors of the manuscripts describing the novel methods. Any disagreements between primary evaluators and authors were debated until general consensus was reached. Once the list of characteristics was completed, data were extracted from pivotal trials, including a summary of the condition, the trial characteristics needed to judge whether the pre-requisites for applying the novel methods could have been fulfilled, and any applicant’s justification for choice of design elements and strategy, if available. We used a two-step approach to determine whether or not the methods could have been applicable and add value:

- Step 1. The static step: evaluation of direct applicability without any adjustments to the original setting of the pivotal studies. This evaluation was based on the (methodological) pre-requisites of the methods and whether these were fulfilled for the trials.
- Step 2. The dynamic step: evaluation of applicability allowing for adjustments to the original setting or design of the studies without changing the original objective and context of the development plan. Changes were made checking therapeutic guidelines, regulatory guidelines or any published article of a study in the same condition, to justify the applicability and improve the drug development program ([24–49]).

For example, a secondary outcome could have been promoted to a primary outcome, or primary and secondary endpoints could have been defined as multiple co-primary endpoints, if this was clinically and methodologically appropriate and sound from a regulatory point of view.

### Analysis, interpretation and synthesis of the results

Based on the comparison between the methods pre-requisites and the characteristics of the pivotal trials, we used applicability evaluation decision trees (Additional file 1: Appendix 2) to measure how applicable the methods were for each EPAR (‘applicable’ denoted by green colour, ‘may be applicable’ denoted by light green colour, ‘no applicability’ denoted by orange colour, ‘no possibility for application irrespective of changes’ denoted by grey colour), depending on pre-requisites fulfillment.

For step 1 if one of the pre-requisites was not fulfilled then non-applicability was concluded, while for step 2 if pre-requisites were not fulfilled, then relevant changes were explored before concluding on applicability or non-applicability.

The applicability is summarized numerically and visualized on a heat map for the first (static) and second (dynamic) step of the evaluation. Based on these heatmaps, we derived recommendations on the use of the novel ASTERIX methodologies per cluster of conditions.

## Results

### Selected EPARs for evaluation

We included 24 EPARs at first, across the six clusters from the public EMA website [21]. As a result of the detailed evaluation, 2 EPARS were re-classified from the cluster chronic: progressive, multiple systems/organs to the cluster of conditions with acute recurrent episodes. Two EPARs were added to ensure at least 4 EPARs per cluster, leading to 26 EPARs in total (Table 2). There was no available OMP and corresponding EPAR for an ultra-rare condition within the cluster of ‘chronic staged conditions’, therefore none could be selected for this cluster.

**Table 2:** EPARs included in the evaluation

Cluster	Drug	Date opinion	Rare or ultra-rare*	Repurposed/ new drug?
Acute: single episodes				
Antracycline extravasation	Savene	2006	Ultra-rare	New drug
Patent ductus arteriosus	Pedea	2009	Rare	Repurposed
Hepatic venooclusive disease	Defitelio	2013	Rare	New drug
Tuberculosis	Sirturo	2014	Rare	New drug
Acute: recurrent episodes				
Cryopirine periodic syndromes	Ilaris	2009	Ultra-rare	New drug
Gram negative lung infection in cystic fibrosis	Cayston	2009	Rare	Repurposed
Narcolepsy	Xyrem	2007	Rare	New drug
Dravet syndrome	Diacomit	2009	Rare	New drug
Sickle cell disease	Sicklos	2007	Rare	New drug
Systemic sclerosis	Tracleer	2009	Rare	New drug
Chronic: stable/slow progression				
Short bowel syndrome	Revestive	2012	Rare	New drug
Adrenal insufficiency	Plenadren	2011	Rare	Repurposed
Thrombocytemia	Xagrid	2009	Rare	New drug
Deficit of lipoprotein lipase	Glybera	2012	Ultra-rare	New drug
Chronic: progressive, one system/organ				
Nocturnal Paroxysmal haemoglobinuria	Soliris	2009	Rare	New drug
Wilson’s disease	Wilzin	2006	Rare	New drug
Congenital errors of bile synthesis	Orphacol	2013	Ultra-rare	Repurposed
Gastrointestinal stromal tumours	Glivec	2009	Ultra-rare	Repurposed
Chronic: progressive, multiple systems/organs				
Fabry disease	Fabrazyme	2008	Ultra-rare	New drug
Cystic fibrosis	Kalydeco	2013	Rare	New drug
Familial amyloid polyneuropathy	Vyndaqel	2011	Rare	New drug
Gaucher disease	Zavesca	2009	Rare	New drug
Chronic: staged condition				
Renal carcinoma	Afinitor	2009	Rare	Repurposed
Pulmonary hypertension	Opsumit	2014	Rare	New drug
Indolent non-Hodgkin lymphoma	Litak	2006	Rare	New drug
Myelodysplastic syndrome	Revlimid	2008	Rare	New drug

*EPAR* European Public Assessment Report

* Rare if prevalence = or > 5/10.000 and > 0.1/10.000 inhabitants; Ultrarare if prevalence = or < 0.1/10.000 inhabitants

### Potential applicability of methods and advantages based on information from actual trials

**Fig. 1:**
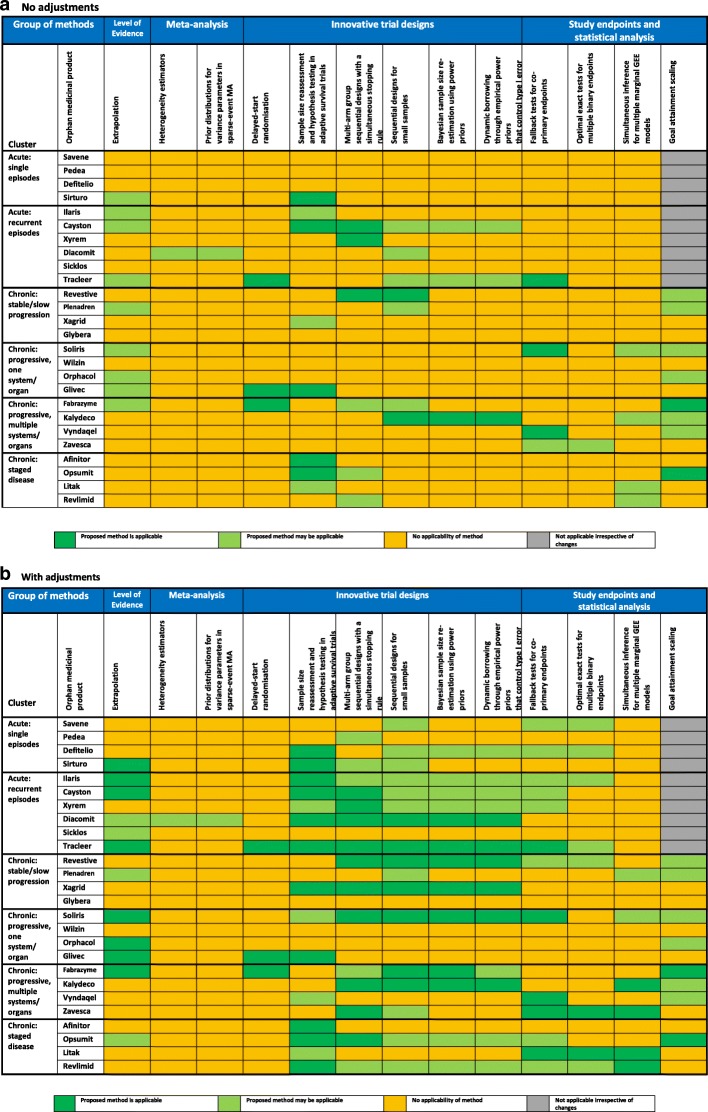
Header: Percentage of EPARs where the methods are applicable

In the first, static step, we found that all individual methods were directly applicable to a minimum of 1 (4%) up to 9 (35%) of the 26 EPARs, and overall each method was applicable to a minimum of 1 (17%) and a maximum of 5 (83%) of the 6 clusters. In the second, dynamic step we found the individual methods applicable in 1 (4%) up to 17 (65%) of the EPARs, and a minimum of 1 (17%) out of 6 and a maximum of 6 (100%) of the 6 clusters (Tables 1 and 3, and Fig. 1).

**Table 3:** Percentage of EPARs where the methods are applicable

METHOD	Applicability in percentage of EPARs			
Step	Static step 1 (no adjustments)		Dynamic step 2 (adjustments)	
Statistic	Percentage of EPARs (*n*/26)	Percentage of clusters (*n*/6)	Percentage of EPARs (*n*/26)	Percentage of clusters (*n*/6)
Extrapolation	35% [9/26]	83%	46% [12/26]	100%
Heterogeneity estimators	4% [1/26]	17%	4% [1/26]	17%
Prior distributions for variance parameters in sparse-event meta-analysis	4% [1/26]	17%	4% [1/26]	17%
Delayed-start randomisation	13% [3/26]	50%	12% [3/26]	50%
Sample size reassessment and hypothesis testing in adaptive survival trials	35% [9/26]	83%	58% [15/26]	100%
Multi-arm group sequential designs with a simultaneous stopping rule	23% [6/26]	67%	58% [15/26]	100%
Sequential designs for small samples	31% [8/26]	67%	66% [17/26]	100%
Bayesian sample size re-estimation using power priors	12% [3/26]	33%	50% [13/26]	100%
Dynamic borrowing through empirical power priors that control type I error	15% [4/26]	33%	50% [13/26]	100%
Fallback tests for co-primary endpoints	15% [4/26]	50%	50% [13/26]	100%
Optimal exact tests for multiple binary endpoints	4% [1/26]	17%	31% [8/26]	83%
Simultaneous inference for multiple marginal GEE models	19% [5/26]	50%	23% [6/26]	67%
Goal Attainment Scaling	31% [8/26]	67%	31% [8/26]	67%

*EPAR* European Public Assessment Report

### Conditions with single acute episodes

Methods that were applicable following adjustments within this cluster of conditions: ‘extrapolation’ (this method is related to ‘dynamic borrowing through empirical power priors’ with the difference that its purpose is to plan a trial in the target population under the assumption that no data in the source population is available yet (as is the case when a paediatric investigation plan is formulated by EMA)) (1/4 EPARs), ‘sample size reassessment and hypothesis testing’ (2/4 EPARs), ‘multi-arm group sequential design with a simultaneous stopping rule’ (2/4 EPARs), ‘sequential designs for small samples’ (3/4 EPARs), ‘Bayesian sample size re-estimation using power priors’ (1/4 EPARs), ‘dynamic borrowing through empirical power priors’ (1/4 EPARs), ‘fallback tests for co-primary endpoints’ (2/4 EPARs) and ‘optimal exact test for multiple binary endpoints’ (2/4 EPARs).

‘Heterogeneity estimators’ and ‘prior distributions for variance parameters in sparse-event meta-analysis’ were not applicable, as all EPARs either used a single pivotal trial or single-arm trials with non-sparse outcomes. Given the possible use of binary outcomes in this cluster, the methods could become applicable in the case where at least two pivotal trials are available. ‘Delayed-start randomisation’ was not applicable as a placebo arm was not used in any of the EPARs. ‘Goal Attainment Scaling’ was not applicable because it requires previous patient experience with the disease to individualize the goals, as well as follow-up assessments at sustained functional level, thus is not suitable for conditions with acute onset and clinical course. Similarly, ‘Simultaneous inference for multiple marginal GEE models’ was not applicable because due to the acute onset and short clinical course limited to a single episode, in this cluster the measurements were not repeated. This does not totally preclude applicability, as the single episode may be long enough to allow valuable use of repeated measurements.

### Conditions with acute recurrent episodes

Methods that were applicable following adjustments were ‘extrapolation’ (5/6 EPARs), ‘sample size reassessment and hypothesis testing in adaptive survival trials’ (5/6 EPARs), ‘multi-arm group sequential designs with a simultaneous stopping rule’ (5/6 EPARs), ‘sequential designs for small samples’ (5/6 EPARs), ‘Bayesian sample size re-estimation using power priors’ (5/6 EPARs), ‘dynamic borrowing through empirical power priors that control type I error’ (5/6 EPARs), ‘fallback tests for co-primary endpoints’ (4/6 EPARs), ‘optimal exact tests for multiple binary endpoints’ (2/6 EPARs), ‘heterogeneity estimators’ and ‘prior distributions for variance parameters in sparse-event meta-analysis’ (1/6 EPARs), ‘delayed-start randomisation’ (1/6 EPARs). ‘Goal Attainment Scaling’ was not applicable for the same reasons as for the cluster of conditions with acute single episodes. ‘Simultaneous inference for multiple marginal GEE models’ appeared not applicable because in this cluster the measurements were not repeated in the setting as being modelled. However, as with acute single episodes it is likely that the method can be extended to be applicable to the type of repeated assessment that apply for conditions in this cluster.

### Chronic conditions with stable or slow progression

Methods that were applicable following adjustments were: ‘extrapolation’ (1/4 EPARs), ‘sample size reassessment and hypothesis testing in adaptive survival trials’ (1/4 EPARs), ‘multi-arm group sequential designs with a simultaneous stopping rule’ (2/4 EPARs), ‘sequential designs for small samples’ (3/4 EPARs), ‘Bayesian sample size re-estimation using power priors’ (2/4 EPARs), ‘dynamic borrowing through empirical power priors that control type I error’ (2/4 EPARs), ‘fallback tests for co-primary endpoints’ (1/4 EPARs), ‘optimal exact tests for multiple binary endpoints’ (1/4 EPARs), ‘simultaneous inference for multiple marginal GEE models’ (1/4 EPARs). ‘Goal Attainment Scaling’ may be methodologically applicable (2/4 EPARs), but its added value may be limited within this cluster as, at least in the selected examples, there are already available validated patient reported outcomes capturing functionality for all targeted patients.

‘Delayed-start randomisation’ was not applicable as most added value is achieved if there is disease progression during the trial period, and treatments having a lasting response, while the clustering was characterised by relatively stable clinical course with treatments having reversible effects. ‘Heterogeneity estimators’ and ‘prior distributions for variance parameters in sparse-event meta-analysis’ were not applicable since there were few randomised trials and the examples included mostly continuous or non-sparse discrete endpoints.

### Chronic progressive conditions led by one system/organ

Methods that were applicable following adjustments included: ‘sample size reassessment and hypothesis testing in adaptive survival trials’ (2/4 EPARs), ‘multi-arm multi-stage trial with a simultaneous stopping rule’ (1/4 EPARs), ‘sequential design for small samples’ (1/4 EPARs), ‘delayed-start randomisation’ (1/4 EPARs)’, ‘Bayesian sample size re-estimation using power priors’ (1/4 EPARs) and ‘dynamic borrowing through empirical power priors that control type I error’ (1/4 EPARs). ‘Heterogeneity estimators’ and ‘prior distributions for variance parameters in sparse-event meta-analysis’ were not applicable, similarly to other clusters of chronic conditions, due to lack of randomised trials, use of continuous or discrete endpoints, or binary endpoints that were not sparse. Also, ‘optimal exact tests for multiple binary endpoints’ was not applicable due to use of non-binary endpoints (i.e. time-to-event or continuous endpoints).

It is noted that this condition cluster contains two EPARs that were approved based on case series and not on experimental or observational trials. There are several reasons for this path, including the scarcity of patients and condition seriousness and severity, leading to ethical concerns and reluctance to use of placebo. In these cases, for methods to become applicable, the entire drug development program needed to be reshaped. Although technically possible, the judgement on whether or not this could be feasible, was out of the scope of our evaluation. For those isolated instances we concluded on non-applicability of methods.

### Chronic progressive conditions led by multiple system/organs

Almost all methods were applicable in this cluster of conditions to some extent following adjustments: ‘sample size reassessment and hypothesis testing in adaptive survival trials’ (1/4 EPARs). ‘Multi-arm multi-stage trial with a simultaneous stopping rule’ (3/4 EPARs), ‘sequential design for small samples’ (3/4 EPARs), ‘delayed-start randomisation’ (1/4 EPARs)’, ‘Bayesian sample size re-estimation using power priors’ (2/4 EPARs), ‘dynamic borrowing through empirical power priors that control type I error’ (2/4 EPARs) ‘fallback tests for multiple endpoints’(2/4 EPARs), ‘optimal exact tests for multiple binary endpoints’ (1/4 EPARs) and ‘GAS’ (3/4 EPARs). Similarly to other clusters including chronic conditions, ‘heterogeneity estimators’ and ‘prior distributions for variance parameters in sparse-event meta-analysis’ were not applicable due to use of continuous or discrete endpoints, lack of randomised trials or binary endpoints that were not sparse.

### Chronic staged conditions

Methods that were applicable following adjustments were ‘sample size reassessment and hypothesis testing in adaptive survival trials’ (4/4 EPARs), ‘multi-arm multi-stage trial with a simultaneous stopping rule’ (2/4 EPARs), ‘sequential design for small samples’ (2/4 EPARs), ‘Bayesian sample size re-estimation using power priors’ (2/4 EPARs), ‘dynamic borrowing through empirical power priors that control type I error’ (2/4 EPARs), ‘fallback tests for multiple endpoints’ (3/4 EPARs), and ‘simultaneous inference for multiple marginal GEE models’ (2/4 EPARs). ‘GAS’ (1/4 EPARs) was applicable in the only non-oncological condition within this cluster, i.e. pulmonary hypertension. ‘Heterogeneity estimators’ and ‘prior distributions for variance parameters in sparse-event meta-analysis’ were not applicable due to use of continuous or discrete endpoints, lack of randomised trials or use of binary endpoints to measure outcomes that were not sparse.

‘Delayed-start randomisation’ was not applicable as most added value is achieved if there is disease progression during the trial period, and treatments having a lasting response, while the clustering was characterised by staged conditions with treatments having reversible effects.

### Advantages and disadvantages compared to the methods used

Potential advantages of using new methods compared to the methods that were used in the drug development program may include (Table 1): reduction in sample size (depending on method and design), more robust evidence, reduced placebo use and/or exposure to placebo or (in retrospect) inferior treatment, patient involvement in benefit-risk assessment. Potential disadvantages were a sufficient level of evidence, but not overwhelming (regardless of the positive or detrimental effect on patients), extra patients needed in case of variance overestimation compared to frequentist approach, more time- and resource- demanding trials, and increased complexity or increased logistic demand on all involved stakeholders.

The evaluation of methods in the ‘meta-analysis’ group resulted in the conclusion that in the current selection of EPARs the two methods were only applicable in the cluster of conditions with acute recurrent episodes, while in fact the two methods have much more potential for applicability. Given the general preference for types of endpoints other than binary (i.e. continuous or time-to-event), that are generally considered to provide better statistical power and sensitivity to change, the meta-analysis methods were often not directly applicable. However, the methods can easily become applicable depending on the choice of endpoint and development background (i.e. number of trials using the same binary endpoint). The two methods could be taken into account in advance and pre-specified to be used in any development program with more than one randomised trial that measures dichotomous outcomes.

## Discussion

The new methods developed in ASTERIX included new proposals for interim analysis and stopping rules in multi-arm parallel trials, methods for sample-size reassessment, rules to optimise boundaries in group-sequential designs, methods to tune the use of prior information from similar trials in Bayesian analysis, considerations to apply flexibility to the level of evidence, new approaches to analyse multiple endpoints, a patient-centered instrument for heterogeneous functional outcomes and two methods for meta-analysis of sparse binary data. The applicability requirements for the methods included mainly the type of measurement (i.e., binary or continuous variable, single or multiple main end-point, scarcity of data), availability of more than one trial, availability of previous studies with good quality data, the length of time to end-point as compared to the time to complete recruitment, and feasibility of randomised designs. While all methods were applicable to some extent and in total could add value on average in 76% of the condition clusters, they were often not directly applicable to the actual trial design or approaches used during clinical development of the OMP as described in the EPAR. Applicability and added value of novel methods were extended when they were not limited to the actual settings of the study design and considered potential changes to the individual trials within the context of the drug development program, i. e., considering the characteristics of the medical condition and optimising the drug development program, rather than improve the trial as presented in isolation.

Most notable strengths of our research are the fact that we systematically evaluated the applicability of the novel methods in a representative sample of real life examples obtained from EPARs, with input from a multidisciplinary team of experts in epidemiology, statistics, drug development, drug regulation and clinical practice. We used therapeutic guidelines in order to determine if reasonable changes could be made to the actual development plan or trials, such as the possibility to use an additional control arm depending on the seriousness and severity of conditions, or availability of standard of care or best supportive care, or the use of a different type of endpoint. Importantly all considered alternatives kept the primary development objectives intact. Some conditions are rare variants of non-rare conditions (such as Dravet syndrome being a rare and severe variant of epilepsy). Hence, we also checked to see if we could borrow design elements and apply strategies from the more common variant (e.g. epilepsy in case of Dravet) leading to proper justification for the changes made and make our conclusions robust.

This evaluation also has some limitations. Firstly, due to feasibility reasons only 4–6 EPARs were evaluated within each cluster. Although we aimed to select a representative sample of different development approaches within each cluster, the applicability within these EPARs might not be fully generalizable to all conditions and drug development plans within the cluster. If a method turned out not to be applicable to any condition within a cluster, the method may still be applicable to some conditions within this cluster that were not selected for evaluation. The reverse is also true: if the method turned out to be applicable in all included EPARs within a cluster, the method may still not be applicable to all conditions within the cluster. Yet, the exercise in itself showed that a systematic approach including the definition of the applicability pre-requisites, together with the definition of the general characteristics of the medical conditions included in a given cluster, allows guidance to investigators on whether they could consider a given method or not for a certain type of medical conditions. Thus, in each individual case the method’s pre-requisites, advantages, and disadvantages should be thoroughly evaluated for adequacy in the full context of the drug development program. While the exercise of applicability may help to define the best toolbox to consider for a given clinical situation, the implications of the methods may differ between conditions and trials, and it should be judged on a case-by-case basis which one of them is optimal.

A further limitation is that the level of detail reported regarding information needed to determine applicability was often limited (e.g. recruitment rates, study timelines, etc.), making it difficult to make a thorough and fully informed judgment on the (in)applicability of the method because this depended on the judgment regarding what changes were deemed feasible or not.

Additionally, only positive opinions were included in our evaluation, given the lack of accessible information on the negative opinions. The impossibility to include negative opinions could have influenced the applicability of the methods. However, we conjecture that in negative opinions there is probably even more potential for improvement.

This work, which was limited to the European regulatory region, could have included the assessment of other orphan drugs approved in other regions, notably in the US and Japan for instance, in order to cover more orphan conditions. Several factors would hamper this approach. The criteria for designation of OMP in the US do not match the one used in EU (i.e. different prevalence cut-off and including medical devices). Furthermore, detailed data on Japanese clinical developments for OMP were not easily available.

We observed that the novel methods are applicable to real life studies, such as those reported in the EPARs, and that they have the potential to improve clinical drug development for small populations and directly address some of the issues flagged in the ‘Guideline for Clinical Trials in Small Populations’.

Not all challenges reported in EPARs or encountered in trials in rare diseases were covered by the novel methods developed within ASTERIX.

One possible avenue for extending this validation exercise based on studies reported in EPARs would be to add on the novel methods developed in the ASTERIX project other study designs and methods applicable to rare diseases available in the literature as the results here demonstrated that this methods validation exercise works and has potential to be extended.

Further research into methods to address these challenges is needed to improve and optimise drug development to ultimately be able to efficiently develop efficacious and safe treatments for all patients suffering from a rare disease.

## Conclusion

Novel methods developed in ASTERIX include methods for trial design, analysis or meta-analysis of trials in small populations. The 13 developed methods have been found to be applicable to real-life examples, and can potentially improve drug development programs. Achieving optimal added value of these novel methods often requires consideration of the entire drug development program, rather than reconsideration of methods for a specific trial. The novel methods tested were mostly applicable in chronic conditions, and acute conditions with recurrent episodes. The implications of the methods may differ in specific medical conditions, and the systematic assessment as presented may guide selecting the optimal methods on a case-by-case basis.

## Additional file

Additional file 1: Provided in doc file: Supplementary Materials.docx. **Appendix 1:** Data extraction form for EPARs including condition summary and criteria list. **Appendix 2:** Decision tree structure for methods evaluation of applicability. **Appendix 3:** List of characteristics used to build the studies profile. **Figure S1.** Proposed method based on validation exercise. How to determine on the optimal trial design (DOCX 35 kb)

## Abbreviations

ASTERIX: Advances in Small Trials dEsign for Regulatory Innovation and eXcellence
BMK: Biomarker
EMA: European Medicines Agency
EPAR: European Public Assessment Report
FDA: Food and Drug Administration
FP7: Seventh Framework Program
OMP: Orphan Medicinal Product
