# Supplementary material for: Applicability and added value of novel methods to improve drug development in rare diseases
Source: Orphanet J Rare Dis. 2018 Nov 12;13:200. doi: 10.1186/s13023-018-0925-0 (PMC6233569; doi:10.1186/s13023-018-0925-0)
Supplement: Supplementary file 1 — Provided in doc file: Supplementary Materials.docx. Appendix 1: Data extraction form for EPARs including condition summary and criteria list. Appendix 2: Decision tree structure for methods evaluation of applicability. Appendix 3: List of characteristics used to build the studies profile. Figure S1. Proposed method based on validation exercise. How to determine on the optimal trial design (DOCX 35 kb) [file 13023_2018_925_MOESM1_ESM.docx]

**Supplementary materials**

**Appendix 1: Data extraction form for EPARs including condition summary and criteria list**

| **Method group X (A, B, C or D): list name [Innovative trial designs, level of evidence, study endpoints and statistical analysis, and meta-analysis]**  **[Name of OMP] EPAR [list number as the ASTERIX ID]**  **Summary of condition and summary of orphan product**  […]  **Summary main clinical trials**   - **(Co-)Primary endpoint(s) [list]** - **Key secondary endpoints [list]** - **Overall patient exposure [list number]** - **Randomised/enrolled number patients [list number]**   **Summary of how applicable are the methods and what are the adjustments, where it is the case.**   \| **OMP ID** \| **Method** \| **Applicable***  **[with/without adjustments]** \| **Reason**  **[list reasons]**  **[list adjustments if the case]** \| **Applied*** \| **If not fully applied [list reasons]** \| **Advantages of applying the method** \| **Disadvantages of applying the method** \| \| --- \| --- \| --- \| --- \| --- \| --- \| --- \| --- \| \| [list ASTERIX OMP ID] \| Method 1 \|  \|  \|  \|  \|  \|  \| \| Method 2 \|  \|  \|  \|  \|  \|  \| \| Method 3 \|  \|  \|  \|  \|  \|  \| \| Method … \|  \|  \|  \|  \|  \|  \|   **Study profile tailored around the methods and developmental plan context.**  [Insert criteria list here]  **Arguments**  **Group A, B, C or D, method 1: [list method]**  [Representative extract from EPAR relevant for the applicability]  **Group A, B, C or D, method 2: [list method]**  [Representative extract from EPAR relevant for the applicability]  **[Conclusion/Discussion on applicability of methods group X on this EPAR]**  **[Added value of method application]** |
| --- | --- | --- | --- | --- | --- | --- | --- | --- | --- | --- | --- | --- | --- | --- | --- | --- | --- | --- | --- | --- | --- | --- | --- | --- | --- | --- | --- | --- | --- | --- | --- | --- | --- | --- | --- | --- | --- |

**Appendix 2: Decision tree structure for methods evaluation of applicability**

**Step 1**

1. Does the trial/study design fulfil all method pre-requisites? Y/N

1a. If Y, then evaluate the applicability and to what extent (assumptions needed, etc.)

| Assign dark green colour to the corresponding cell if all pre-requisites are fulfilled.  Assign light green colour to the corresponding cell if at least one pre-requisite is challenging/difficult to be fulfilled, but none is impossible to fulfil. |
| --- |

1b. If N*, then check to see if it could have fulfilled the other pre-requisites Y/N upon relevant adjustments

| *Assign orange colour to the corresponding cell if at least one pre-requisite is not possible to fulfil, but it does make sense to consider. |
| --- |

**Step 2** *(taking into account other regulatory guidelines, studies, therapeutic guidelines)*

1b.a. If Y, then evaluate if implementation possible and to what extent

| Assign dark green colour to the corresponding cell if all pre-requisites become (easily) applicable upon relevant adjustments.  Assign light green colour to the corresponding cell if at least one pre-requisite still is challenging/difficult to fulfil even after relevant adjustments, but none is impossible to fulfil. |
| --- |

1.b.b. If N, then stop and conclude on non-applicability

| Assign orange colour to the corresponding cell if at least one pre-requisite is not possible to fulfil even upon relevant adjustments, but is does make sense to consider. |
| --- |

If a method is clearly not applicable by default, grey is assigned (*NB: this only occurred for Goal Attainment Scaling in the acute clusters - single episodes and recurrent episodes*).

**Appendix 3: List of characteristics used to build the studies profile**

| # | Characteristic |
| --- | --- |
| a.1.1. | number of arms in main trial(s) |
| a.1.2. | Interim analysis Y/N, If so, reason: stopping for futility, overwhelming evidence of efficacy, safety. |
| a.1.3. | Type of endpoint (primary endpoint) (binary, continuous)  Composite? Time to event? |
| a.1.4. | Type of (major) secondary endpoints (fill in as above) |
| a.1.5. | Adaptive randomisation? Detail |
| a.1.6. | Delta time= recruitment - assessment (delay) /immediate or delayed response |
| a.1.7. | Recruitment rate |
| a.1.8. | Seamless/adaptive design? |
| a.1.9. | Allocation ratio? |
| a.1.10. | Did they allow dropping of arms? |
| a.1.11. | What was the control group? Add-on? |
| a.1.12. | MRCT? Multicentric? How and how many? |
| a.1.13. | Summary of models used in planning (e.g., disease progression, dropout, dose–response) |
| a.2.1 | t1e control and sample size - Detail |
| a.2.2. | Justification of design (i.e. use or not of control, what control and how?) |
| a.2.3. | (Blinding of) sample size reassessment? |
| a.2.4. | Immediate/Delayed responses +/- recruitment rate |
| a.2.5. | Correction of rejection boundaries (in case of small samples)? |
| a.3.1. | Sequential trial? With a maximum sample size? |
| a.3.2. | Disease severity and seriousness |
| a.3.3. | Available treatment options/Ranking and selection (BSC, SOC, other treatment or strategy?) |
| a.3.4. | Possible toxicity of the treatment under consideration |
| a.3.5. | available sample size/Maximum sample size estimation and consideration (i.e. patient horizon) |
| a.4.1. | Type of endpoint - (continuous, binary) |
| a.4.2. | Sequential design? Can it be designed as sequential? Interim analyses? How many? Detail |
| a.4.3. | Randomisation considerations? Randomised? How? |
| a.5.1. | Control arm? Justification for use/no use |
| a.5.2. | Available data from previous similar study/ies? (estimated variance from prior (pilot) study?) |
| a.5.3. | (Ideally) the same sample size per group in old and new trial? |
| b.1.1 . | Slowly and constantly progressive disease? |
| b.1.2. | Placebo comparator? |
| b.1.3. | Intervention has lasting response/remission? |
| b.2.1. | Is a standard of care/ therapy known? (used too?) Detail |
| b.2.2 | Is there uncertainty in the natural course of the disease (or is it possible to predict progression for each patient with 100% certainty)? |
| b.2.3. | The Sponsor's/Investigator's justification for a non-RCT, if any. Any other possible reasoning? |
| b.2.4. | If SAT(s) then how many SATs? Concomitantly conducted? Reasoning? Detail |
| c.1.1. | Number of subgroups/strata? |
| c1.2. | Mutually exclusive subgroups/strata? |
| c.1.3. | Subgroups of equal size? |
| c.1.4. | Randomisation? Randomised? |
| c.1.5. | Two treatment arms? |
| c.2.1. | PIP needed/waived/deferred. Is it applicable to the setting? PIP subject to Conditional/Exceptional approval? |
| c.2.2. | Belief in prior? Justification Guide how much evidence is needed from what sources |
| c.2.3. | What is your posterior probability that there is indeed a relevant positive treatment effect, after adding evidence to your prior Guide whether or not to continue with another trial or searching for other alternative sources of evidence. |
| c.2.4. | Same underlying mechanism of action, similarity of response to treatment, similar dose-response relationship to conclude the mechanism is translatable to the target population? |
| c.2.5. | Same disease symptoms in adults and children, regarding similarity of disease progression? Determine whether full or partial extrapolation can be done. |
| c.2.6. | How is the timing of the paediatric trial compared to the adult trial? Detail Subsequent/in parallel/overlapping? |
| c.2.7. | Repurposed drug or extension of indication? If completely new drug there cannot be much confidence on extrapolation, no full extrapolation without PIP. |
| c.2.8. | Prior effect size estimate? |
| d.1.1. | Repeated measurements? |
| d.2.1. | More than one primary endpoint? |
| d.2.2. | Co-primary endpoints? Efficacy expected in one of them? |
| d.2.3. | One test per endpoint? |
| d.2.4 | Hierarchical testing? |
| d.2.5. | Co-primary endpoints need to be tested sequentially according to a pre-defined order/ranking? Detail |
| d.2.6. | Number of co-primary endpoints |
| d.3.1. | 2, 3, 4, 5 binary endpoints? Detail |
| d.3.2. | Small sample sizes (1 to 50 per group)? |
| d.3.3. | A priori (optimal) rejection region (defined)? |
| d.3.4. | Prior distribution of effect sizes? Detail |
| d.3.5. | Power averaged over the prior distribution effect sizes? |
| d.3.6. | Correction method? Detail |
| e.1.1. | Are the treatment arms in all studies the same? |
| e.1.2. | Is the endpoint dichotomous? |
| e.1.3. | Number of studies? |
| e.1.4. | Are all studies of equal size? |
| e.2.1. | Are there at least two randomized, controlled trials available? |
| e.2.2. | Same primary endpoint? Primary endpoint in one of the trials used as key secondary in the other trial(s)? Primary endpoint/key secondary endpoint in one of the trials used as co-primary in the other trial(s)? |
| e.2.3. | Sparse events? |
| e.2.4. | Supportive studies similar with the pivotal trial? |
| e.2.5. | Treatment effect size estimate? Provided? Clearly? Detail |
| e.2.6. | Are the patients equally allocated per study? |
| f.1.1. | GAS used? |
| f.1.2. | Primary endpoint relevant for the entire array of patients? |
| f.1.3. | Heterogeneous disease course/heterogeneous population or unstable baseline? |
| f.1.4. | Any relevant PRO (i.e. HRQoL)? Mixed with clinician input? Carer input? Detail |
| f.1.5. | Details about validation (validated PRO?) Detail |
| f.1.5. | Patient involved in the design of PRO? Designed by clinician/patient per individual? |
| f.1.6. | Is the measurement at functional level relevant? |

**Supplementary Figure 1. Proposed method based on validation exercise. How to determine on the optimal trial design.**
